# Investigation of Film with β-Galactosidase Designed for Stabilization and Handling in Dry Configuration

**DOI:** 10.3390/molecules200917180

**Published:** 2015-09-18

**Authors:** Liguang Zhang, Andrew Otte, Min Xiang, Dexiu Liu, Rodolfo Pinal

**Affiliations:** 1 Department of Industrial and Physical Pharmacy, Purdue University, West Lafayette, IN 47907, USA; E-Mails: ivy0714@126.com (L.Z.); aotte@purdue.edu (A.O.); 2 College of Pharmacy, Suzhou Health College, Suzhou 215009, China; E-Mails: xiangmin99@126.com (M.X.); dxliu@szhct.edu.cn (D.L.)

**Keywords:** gelatin-based film, β-galactosidase, protein stabilization, cold chain, dry protein

## Abstract

Gelatin-based films with an immobilized enzyme designed for extending the stability of the protein in dry, non-powder configuration with precise dosing attributes were subjected to stress conditions of temperature and relative humidity. β-galactosidase was used as model functional protein. The film configuration preserved the activity of the enzyme under the different storage conditions investigated, which include room temperature under low (ambient) and high (75%) relative humidity, and 36 °C under low (oven) and high relative humidity conditions for a period of 46 days. The influence of the enzyme and plasticizer (glycerol) on the physical and mechanical properties of the films was investigated using DMA (dynamic mechanical analysis). Films containing 5% β-galactosisdase and glycerol concentrations of 14% or greater exhibited greater tensile strength, Young’s modulus, and elongation at break than films with equal concentrations of plasticizer but devoid of any enzyme. The surface texture of the films was analyzed using scanning electron microscopy (SEM). β-galactosidase and glycerol have opposite effects on the surface morphology of the films. Increasing concentrations of the enzyme result in rougher film surface, whereas increasing the concentration of glycerol leads to films with denser and smoother surface. The results obtained suggest that the dry film configuration approach can help in facilitating the stabilization, handling, storage, and transportation of functional proteins in a cost effective manner.

## 1. Introduction

Recent and rapid advances in biotechnology place proteins at the forefront of drug therapy, leading to a significant increase in the number of approved protein based drug products. Owing to their high specificity and bioactivity, proteins have become the drugs of choice for the treatment of numerous diseases [1,2]. Among their advantages is the fact that proteins cause fewer side effects and possessing great potential to cure diseases instead of merely relieving their symptoms [3]. Enzymes are proteins with biological catalytic functions and can catalyze all reactions in the body in very small amounts. In addition, due to their high efficiency and specificity, enzymes are increasingly becoming an important part of chemical synthesis [4,5] and are widely used in the industrial biotechnology [6].

Despite their many advantages, a universal issue of concern regarding proteins is their stability. Proteins often have labile bonds and numerous chemically reactive groups on their side chains, which could be readily modified, leading to loss of activity [7,8,9]. In addition, proteins are highly sensitive to environmental conditions, such as temperature and pH. Variation in these conditions may lead to denaturation and/or aggregation of proteins [10,11,12,13]. The challenges posed by protein stability are not limited to chemical changes. The physical stability of proteins during processing, storage, transportation, and use is also a concern [14,15,16].

There are several strategies for stabilizing enzymes, such as the use of additives, chemical or genetic modification and by immobilization. Among these approaches, immobilization is most widely applied [17] especially in industrial bioprocesses in recent years. Immobilization can locate the enzyme in a specific area in a sensor where the transducer is placed [18]. Immobilization has the potential for the re-use of enzymes. In addition, immobilization can further improve enzyme properties due to multipoint attachment, generation of favorable environment, prevention of aggregation or proteolysis, *etc*. [19,20,21,22,23,24]. Immobilization may also improve other enzyme properties, like specificity, or even activity [25,26,27].

It is very important to understand how proteins can be stabilized and to find ways of increasing their stability in order to fully exploit the many advantages that such macromolecules offer. In this work, we investigate the use of gelatin films as a means of preserving proteins in a dry, but stable, state. We use β-galactosidase as model protein and immobilized it in the gelatin-based film, then monitor the enzyme activity to assess protein integrity in the film configuration and investigate the ability to recover enzyme activity from films stored under different conditions.

## 2. Results and Discussion

### 2.1. Stability of β-Galactosidase in the Films

The β-galactosidase family comprises a number of proteins whose enzymatic activity varies, depending on testing conditions such as pH, temperature and buffer system, as well as on the origin of the enzyme. Since *o*-nitrophenyl-β-d-galactopyranosides (ONPG) is a common substrate for different β-galactosisdases, the colored product, *o*-nitrophenol (ONP) from the reaction between β-galactosisdase and ONPG has made the latter the most widely-used indicator for the enzymatic activity of this family of proteins. In general, the optimum pH conditions for β-galactosidase is in the range of pH of 7–8. Using ONPG, β-galactosidase from *Kluyveromices fragilis* and phosphate buffer with mercaptoethanol, Santos *et al*. [28] reported pH 7 as the optimum value for enzyme activity. A pH of 7 was also reported as optimum for β-galactosidase from *Lactobacillus delbrueckii* in PBS [29]. For β-galactosidase from *E. coli*, an optimum pH range of 7.5–8 was found in PBS enriched with mercaptoethanol and magnesium [30]. However, optimal pH conditions as low as 4.5 [31] and 2.8 [32] have been reported. A feasible explanation for the wide range of optimal pH values reported lies on the dual effect of pH with the use of ONPG, as discussed below.

**Figure 1:**
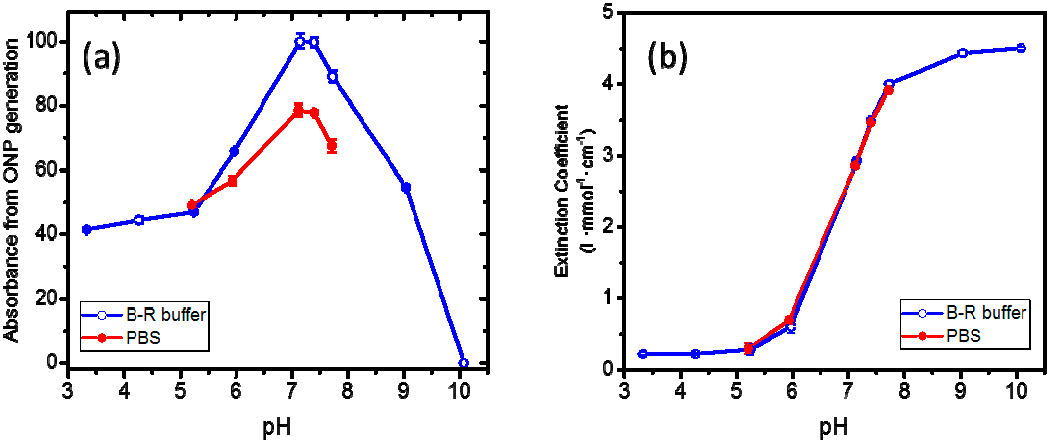
(**a**) Relative uncorrected absorbance (420 nm) of ONP generated by the reaction of ONPG with β-galasctosidase in solution as a function of pH in Britton-Robinson (B-R) and phosphate (PBS) buffers. Absorbance in pH 7.15 B-R buffer is used as reference; (**b**) Extintion coefficient of ONP (420 nm) in solution as a function of pH in Britton-Robinson (B-R) and phosphate (PBS) buffers. Lines are shown as visual aids.

The dual effect is as follows. On the one hand, the enzyme activity is affected by the pH conditions. On the other hand, the pH also affects the extinction coefficient (molar absorptivity) of ONP, whose spectrophotometric quantification serves as indicator of enzyme activity. Figure 1a shows the activity of β-galactosidase as a function of pH in two different buffers, without correction for the effect of pH on the absorbance of ONP. In other words, the activity values shown in the graph are based on direct absorbance readings (normalized to the highest absorbance value) for ONP. Figure 1b shows the extinction coefficient of ONP at 420 nm as a function of pH. The extinction coefficient values in Britton-Robinson (B-R) and phosphate (PBS) buffers are essentially superimposable. This indicates that the differences between the two plots in Figure 1a are the result of the effect of the buffer on enzyme activity and not of different effect of pH on the extinction coefficient of ONP resulting from the buffer choice. However, for any given pH value, the difference in enzyme activity due to the buffer does not directly correspond to the difference between the respective plots in Figure 1a. The reason is that in the same pH range, while the absorbance readings of ONP generated from enzyme activity (Figure 1a) first increase and then decrease, the extinction coefficient of ONP (Figure 1b) undergoes a gradual increase throughout. The net result is that equal concentrations of ONP at different pH would give a different absorbance reading, depending on the specific pH conditions.

**Figure 2:**
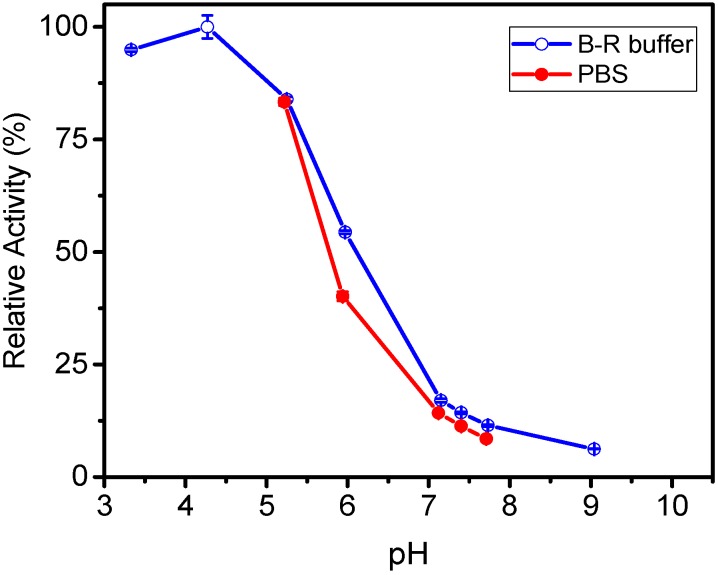
Relative activity of β-galactosidase as a function of pH in Britton-Robinson (B-R) and phosphate (PBS) buffers. The enzyme activity was assessed by applying the correction for absorbance (420 nm) resulting from the effect of pH on the extinction coefficient of ONP (see Figure 1b). Activity in pH 7.15 B-R buffer is used as reference. Lines are shown as visual aids.

Figure 2 shows the activity of β-galactosisdase based on ONP absorbance values corrected for the effect of pH on the extinction coefficient. The enzyme activity exhibits a similar profile as a function of pH with the two buffers. However, the B-R buffer elicits a somewhat higher activity on the enzyme. The figure shows that maximal activity was obtained in the pH range of 4–5. The results in Figure 1 and Figure 2 show that when comparing the activity of β-galactosidase under different pH conditions, it is necessary to make the appropriate correction for the effect of pH on the extinction coefficient of ONP. If the measurements are made all under the same pH conditions, the correction is not strictly necessary since the scaling of all absorbance values is the same. In this report, all measurements of β-galactosidase activity were conducted at pH = 7.4. In addition, since PBS is routinely used in many screening applications, this buffer was chosen as the working buffer for assessing the activity of β-galactosidase in the film configuration in the study.

We should point out that the activity of bulk β-galactosisdase can be maintained for prolonged periods if the powder is kept in a tightly-sealed container under controlled temperature conditions. Monitoring the stability of the bulk, raw material enzyme powder is not within the scope of this investigation. The objective of this study is to explore the stability of the enzyme as a dry, non-powder functional product, with precisely-known dosing attributes. Films provide such a functional configuration, and that is the focus of the study presented here.

**Figure 3:**
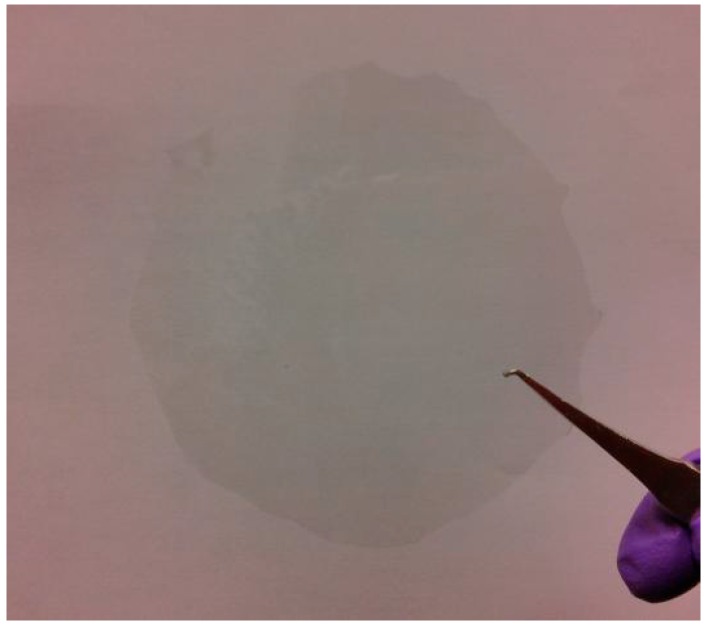
The film prepared for assaying.

**Figure 4:**
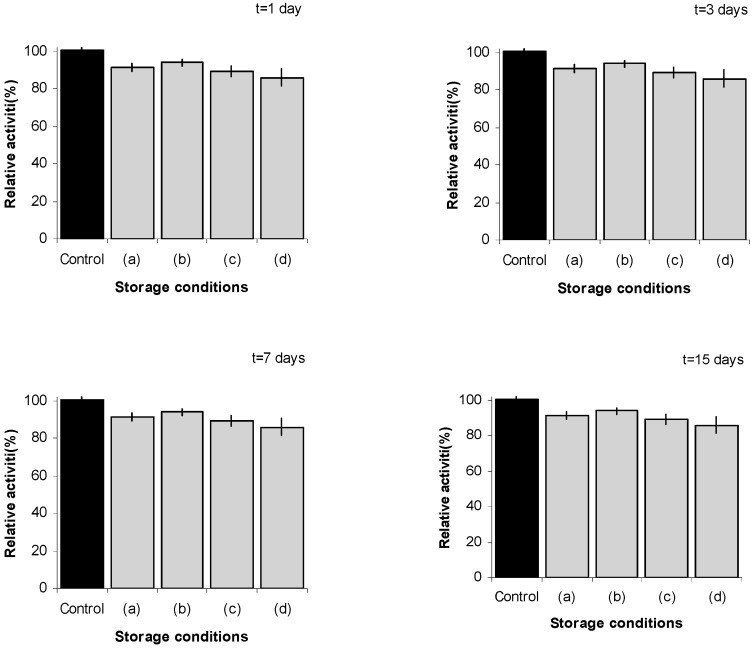
Activity of β-galactosidase immobilized in dry film configuration under different storage conditions. Control: freshly prepared solution. Activity of control solution is used as reference. (**a**) room temperature (23 ± 2 °C)+ 55% RH; (**b**) room temperature and 75% RH; (**c**) 36 °C (dry environment); and (**d**) 36 °C and 75% RH.

A photograph of the type of films prepared in this investigation is shown in Figure 3. The films containing the enzyme were stored under in different conditions: (a) room temperature (23 ± 2 °C) + 55% relative humidity (RH); (b) room temperature +75% RH; (c) 36 °C; (d) 36 °C +75% RH and (e) under refrigeration (7 ± 2 °C). The activity of enzyme embedded in the films was tested at different time points for a period of 46 days. For the activity test, the films containing the enzyme were dissolved in PBS (pH = 7.4) and the activity of the enzyme was tested with ONPG in solution as the substrate. Figure 4 shows the stability results for β-galactosisdase in film configuration for a period of 15 days. There is a moderate decrease in activity from enzyme recovered from the films. However, it is noteworthy that even under stress stability conditions (those involving 75% RH), the enzyme activity is maintained very much at the same level as when kept under the milder storage conditions of low humidity. These results suggest that the film configuration offers a robust protective environment for maintaining enzyme activity in a dry, simple to handle, non-powder configuration.

**Figure 5:**
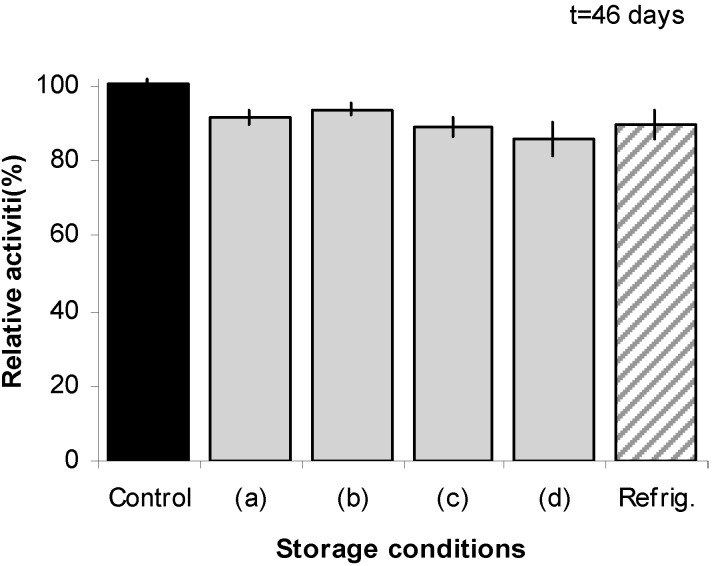
Stability of β-galactosidase immobilized in dry film configuration after 46 days under different storage conditions. Control: freshly prepared enzyme solution. Activity of control solution is used as reference. (**a**) Room temperature (23 ± 2 °C)+ 55% RH; (**b**) room temperature and 75% RH; (**c**) 36 °C (dry environment); and (**d**) 36 °C and 75% RH, Refrig.: dry film with enzyme stored under refrigeration.

Figure 5 shows the stability results using the enzyme after a period of 46 days. The reference refrigerated dry film undergoes a modest decrease in enzyme activity. Comparatively, the figure also shows that the same level of enzyme activity is maintained, without the need of refrigeration, when the enzyme is loaded into the films. These results indicate that the film configuration offers a viable and practical means of maintaining the integrity of the enzyme while in dry state, that also protects during prolonged exposure to high humidity conditions and high ambient temperature. These findings are promising in the sense that they are likely to help address some of the issues pertaining to the so-called cold chain. It is considerably more expensive to keep products at 2–8 °C (refrigeration) or at frozen temperatures, than it is to keep them under ambient or uncontrolled conditions [33]. The cold chain, which refers to the management (storage and transportation) of temperature-sensitive products as they move through the supply chain, is an aspect of significant and increasing importance to protein-based products, such as biopharmaceuticals [34]. In particular, transportation is considered the weakest link in any drug supply chain [35]. The results in Figure 4 and Figure 5 suggest that preservation of functional proteins in dry films could provide an effective and cost-effective means of protection against ambient temperature and humidity for the storage, handling, and transportation of protein-based products.

### 2.2. Mechanical Properties

One additional advantage of using the film configuration for preserving the enzyme is that it makes it a rather simple task to collect specific amounts or doses of the protein. Since the enzyme is present at a known concentration in the film, collection of a precise amount of the enzyme can be readily achieved by cutting a portion of the specified area. However, for the film configuration to be a viable means for handling (and transportating) the dry enzyme, beyond the preservation of activity, it is important that the films have the appropriate mechanical characteristics. To this effect, the mechanical properties of the films were investigated.

The presence of an enzyme in protein-based films has been found to have an effect on the mechanical properties of the films. Mariniello *et al*., found that in pectin-soy protein films, tensile strength values of the films obtained in the presence of transglutaminase (TGase) were two times greater than in films prepared without enzyme, whereas elongation at break (ε_b_) decreased by as much as one half [36]. In the present study, concentrations of β-galactosidase exceeding about 5% (*w*/*w*) rendered films that were too brittle, hence too fragile, to readily handle. However, from a film handling perspective, we also found that at enzyme concentrations below the ~5% threshold, the presence of β-galactosidase resulted in improved mechanical properties of the films, as discussed below.

**Figure 6:**
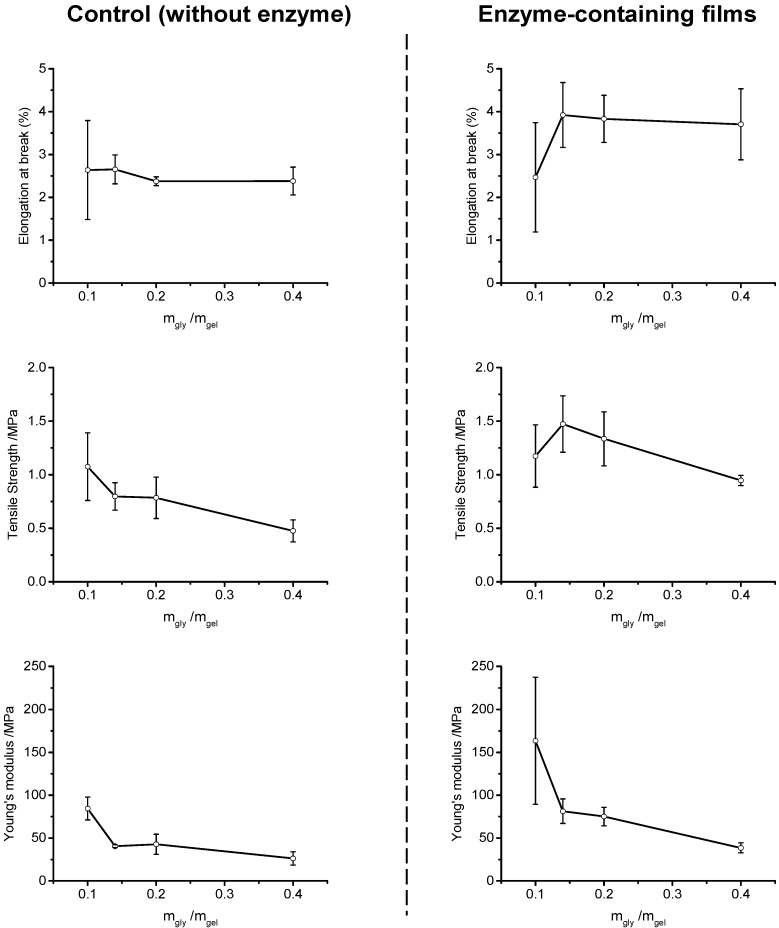
Mechanical properties of films as a function of glycerol concentration used as plasticizer. **Left**: control films, enzyme free. **Right**: films containing 5% β-galactosidase. Lines are shown as visual aids.

Plasticizers are an important component of virtually all polymer films. They make it possible to change and control the physico-mechanical properties of films without changing the basic chemistry involved. Plasticizers work by affecting intermolecular forces and increase the mobility of biopolymer chains [37]. Glycerol is commonly used as a plasticizer of biocompatible polymers of different types, including those of carbohydrate, protein, and synthetic origin. Glycerol provides a biocompatible and effective means for manipulating the physical properties of polymeric materials [38]. Figure 6 shows the mechanical properties of the films as a function of plasticizer (glycerol) concentration, relative to gelatin, represented as the ratio of glycerol to gelatin (m_gly_/m_gel_) in the horizontal axis. The left side of the figure shows the mechanical properties of control (or placebo) films, *i.e.*, films devoid of any β-galactosidase. The right side of the figure shows the effect of the presence of the enzyme on the mechanical properties of the films. Figure 6 shows that at the low plasticizer concentration (m_gly_/m_gel_ = 0.1), the presence of the β-galactosidase in the films has a negligible effect on ε_b_. However, while ε_b_ remains essentially constant as a function of plasticizer concentration in the control films, an increase in ε_b_ is observed at m_gly_/m_gel_ = 0.14 in the enzyme-containing films, followed by a plateau.

Increasing plasticizer concentration results in a continuous decrease in the tensile strength (σ_b_) of the control films. Conversely, in the films containing β-galactosidase, an initial increase in σ_b_ is initially observed, before the continuous decrease typically observed with polymers as the concentration of plasticizer increases. These results provide some clue into the type of interaction between gelatin and β-galactosidase in the films. The increase in mechanical strength with increasing plasticizer concentration exemplify antiplasticization, a phenomenon commonly encountered in polymeric systems [39]. The occurrence of antiplasticization has been associated with a net reduction in the free volume in the polymer-plasticizer mixture [40]. The results in Figure 6 suggest that the combined effects of glycerol and β-galactosidase reduce the internal hydrogen bonding between gelatin-gelatin chains [41]. Hydrogen bonding is a highly directional type of interaction and its reduction leads to a shortening distance (reduced free volume) between the molecules [42]. The presence of plasticizers at the appropriate concentrations increase the flexibility and workability of biopolymer films. This is often a desirable situation since plasticizers affect several physico-mechanical properties. In some cases, however, plasticizers can also exert non desirable changes on the films. The results in Figure 6 reflect the effect of the use of a single plasticizer. Thomazine *et al*., found that the use of plasticizer blends can be exploited as a means to better control the properties of gelatin films [43]. Therefore, it should be possible to optimize (if needed) the mechanical properties of gelatin films containing β-galactosidase.

Figure 6 shows that the presence of β-galactosidase in the films results in an increase in Young’s modulus (*E*) at low glycerol concentrations. The Young’s modulus values reported correspond to the maximum of the derivative of stress (MPa) *vs.* strain (%) recorded by the instrument. Increasing the plasticizer concentration decreases *E* in both the control and enzyme-containing films. The decrease in *E* with glycerol concentration is more pronounced in the enzyme-containing films, such that at the higher plasticizer concentration (m_gly_/m_gel_ = 0.4), the two types of film exhibit similar *E* values.

### 2.3. Surface Morphology

**Figure 7:**
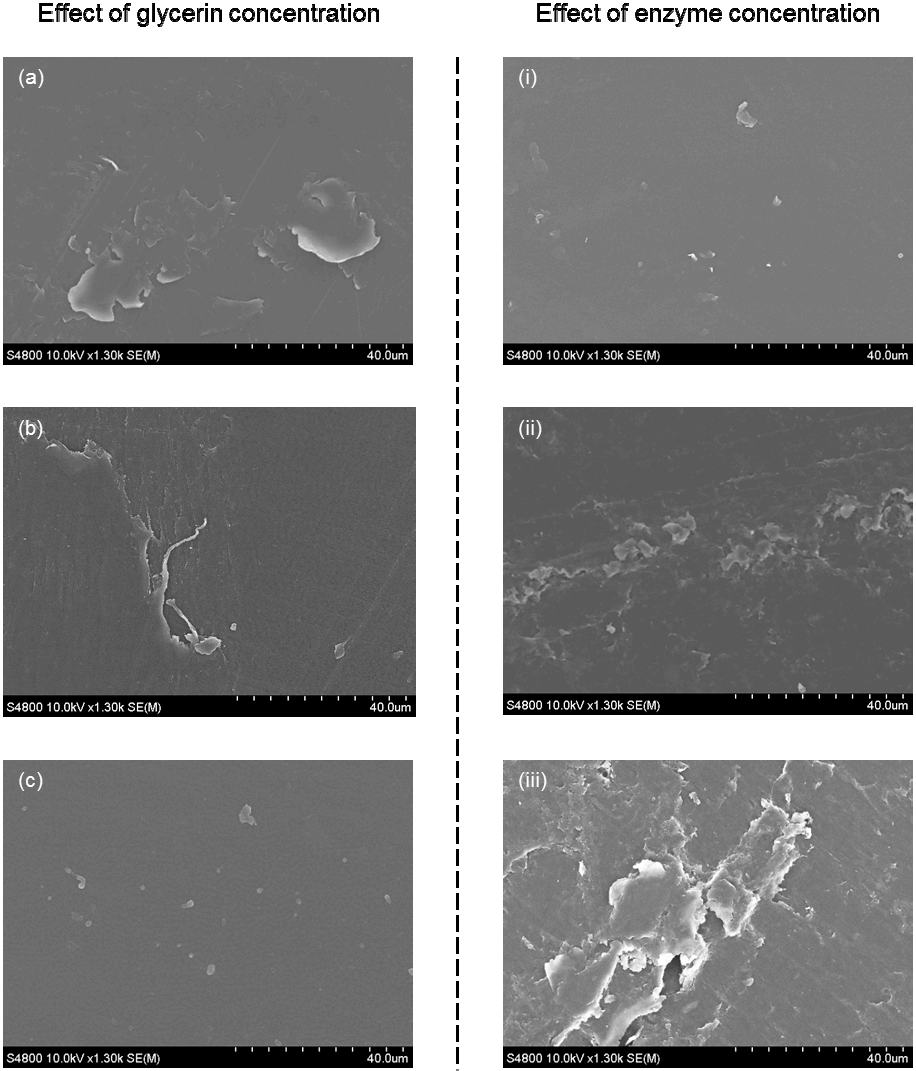
Surface morphology of films containing. Effects of increasing concentration of glycerol used as a plasticizer (**left**) and increasing concentration of β-galactosidase (**right**). **Left**: (**a**) 10% glycerol; (**b**) 14%; and (**c**) 20%. **Right**: (**i**) 3.5% β-galactosidase; (**ii**) 8%; and (**iii**) 10%.

The effects of both plasticizer and β-galactosidase concentration on the films leave similar physical marks on the films. However, the effect of the two variables work on opposite direction. Figure 7 presents scanning electron microscopy (SEM) images showing the physical characteristics observed with different concentrations of glycerol and β-galactosidase. The left hand side of the figure shows the surface characteristics obtained with increasing concentration of glycerol in films with a fixed content of β-galactosidase of 5%. The right hand side of the figure shows the effect of increasing concentrations of β-galactosidase on the surface of films containing a fixed concentration of 10% glycerol. The surface morphology of the films is smoother and of denser appearance with increasing concentration of glycerol and with decreasing concentration of β-galactosidase.

Li *et al*., reported gelatin films without enzyme (tyrosinase) as homogeneous, whereas gelatin film containing immobilized tyrosinase as having a bumpy structure [44]. Figure 7 shows that the presence of β-galactosidase has a similar effect on glelatin films. These observations indicate that optimization of the mechanical and physical properties of gelatin films containing immobilized enzymes resides, to a large extent, on finding the right balance between enzyme and plasticizer content.

## 3. Experimental Section

### Material and Methods

The enzymatic activity of β-galactosidase from *Aspergillus oryzae* was assessed by using ONPG as substrate. The enzyme, substrate, ONP, type A gelatin (100 Bloom from porcine skin), as well as glycerol were purchased from Sigma Chem. Co (St. Louis, MO, USA). Phosphoric, acetic, and boric acids, used for preparing buffer solutions, were purchased from Macron (Center Valley, PA, USA). Unless otherwise specified, chemicals and reagents were used as received. The reaction of β-galactosidase with ONPG produces the colored product ONP [28,45]. The intensity of color produced by the enzymatic reaction was measured using UV-VIS spectrophotometry (Varian Cary 300 bio) at 420 nm. The absorption coefficient of ONP was obtained by 10 μg/mL ONP in the buffer of different pH at 420 nm.

The phosphate buffer solution (PBS) used in this work was 10 mM pH 7.4 (NaCl 8.01 g·L^−1^, KCl 0.20 g·L^−1^, Na_2_HPO_4_·2H_2_O 1.78 g·L^−1^, and KH_2_PO_4_ 0.27 g·L^−1^). A range of pH of 5.2–7.7 PBS was obtained by adjusting the former solution to the required pH with HCl or NaOH. The Britton-Robinson (B-R) buffer used in the study contained 20 mM of each, phosphoric, acetic, and boric acid. The B-R buffer employed covered the pH range from 3.3–10.0, adjusted by adding HCl or NaOH, as required.

All films were prepared by solvent casting. 2.5 g Gelatin powder was mixed with 50 mL deionized distilled water using different amounts (wt %) of glycerol as plasticizer. The gelatin and glycerol were mixed in aqueous solution and heated at 36 °C for 2 h. Different amounts (wt %) of enzyme (10.3 units/mg) were then added to the gelatin solution while applying gentle stirring. The same type of gelatin solutions, but devoid from any enzyme (control), were used as reference. Gelatin solutions (for control), as well as those containing the enzyme, were uniformly cast on petri dishes (100 mm diameter) and left undisturbed at room temperature (23 ± 2 °C and 55% RH) for 2 days. The ensuing congealing and evaporation produced the films used in the study. The obtained films were manually peeled off and used in the subsequent analysis. The thickness of film was measured using a digital caliper (Marathon Watch Company Ltd. Richmond Hill, ON, Canada).

Micrographs were obtained using a Hitachi 4800 SEM instrument. All the samples were examined using an accelerating voltage of 10.0 kV. Dynamic mechanical analysis (DMA) measurements were performed with a Q800 DMA (TA Instruments). The films were conditioned at 25 °C and 55% RH for 48 h prior to mechanical testing. The film strips for DMA analysis were cut from the cast film (typically 0.3 cm × 2.0 cm). The tensile strength (σ_b_, MPa) and elongation at break (ε_b_, %) were obtained directly from the stress *vs.* strain curve.

## 4. Conclusions

We have demonstrated a gelatin-based film capable of stabilizing β-galactosidase in dry form. The activity of the enzyme in the dry film can be maintained for a prolonged period of several weeks even under conditions of constant exposure to high temperature and high humidity. The remarkable ability of the gelatin films to protect the enzyme by preserving its activity under stress stability conditions points toward the possibility of stabilizing enzymes and other therapeutic proteins in a readily-applicable manner. The crosslinking of these types of films may be exploited to convert this reversible immobilization method into a means for obtaining an immobilized biocatalyst. In addition, stabilization by the dry film approach has the potential to significantly facilitate the handling, storage, and transportation of protein-based products that are becoming an ever-more important part of health care efforts worldwide. Inclusion of the functional protein into films plays an important role on the physical and mechanical properties of the resulting film, thus affecting the critical attributes necessary for handling and transportation. In the example presented in this study, the presence of the enzyme in the film changes the mechanical properties of the films in manner that is favorable for handling and transportation. However, the obtained results indicate that even if that were not the case, simple formulation changes, such as adjustment of the levels of plasticizer used, could be used to optimize the physical and mechanical properties of the films. The use of this type of films with immobilized enzyme maybe further exploited in reactions in anhydrous media, when a protein solution is not feasible. In short, the film with β-galactosidase showed excellent stability, as well as physical and mechanical properties, and offers a wide range of applicability. These types of results are expected to apply to other bioactive proteins.

## References

[1] Frokjaer S., Otzen D.E. (2005). Protein drug stability: A formulation challenge. Nat. Rev. Drug Discov..

[2] Park K., Kwon I.C., Park K. (2011). Oral protein delivery: Current status and future prospect. React. Funct. Polym..

[3] Morishita M., Peppas N.A. (2006). Is the oral route possible for peptide and protein drug delivery?. Drug Discov. Today.

[4] De Gonzalo G., Orden A.A., Bisogno F.R. (2012). New trends in organic synthesis with oxidative enzymes. Curr. Org. Chem..

[5] Schmid M., Sängerlaub S., Wege L., Stäbleret A. (2014). Properties of Transglutaminase Crosslinked Whey Protein Isolate Coatings and Cast Films. Packag. Technol. Sci..

[6] Sulaiman S., Mokhtar M.N., Naim M.N., Baharuddin A.S., Sulaiman A.A. (2015). Review: Potential usage of cellulose nanofibers (CNF) for enzyme immobilization via covalent interactions. Appl. Biochem. Biotechnol..

[7] Putney S.D. (1998). Encapsulation of proteins for improved delivery. Curr. Opin. Chem. Biol..

[8] Putney S.D., Burke P.A. (1998). Improving protein therapeutics with sustained-release formulations. Nat. Biotechnol..

[9] Hammann F., Schmid M. (2014). Determination and Quantification of Molecular Interactions in Protein Films: A Review. Materials.

[10] Manning M., Patel K., Borchardt R. (1989). Stability of Protein Pharmaceuticals. Pharm. Res..

[11] Wang W. (1999). Instability, stabilization, and formulation of liquid protein pharmaceuticals. Int. J. Pharm..

[12] Haque M.A., Chen J., Aldred P., Adhikari B. (2014). Drying and denaturation characteristics of whey protein isolate in the presence of lactose and trehalose. Food Chem..

[13] Haque M.A., Putranto A., Aldred P., Chen J., Adhikari B. (2013). Drying and Denaturation Kinetics of Whey Protein Isolate (WPI) During Convective Air Drying Process. Dry Technol..

[14] Tavakoli-Keshe R., Phillips J.J., Turner R., Bracewell D.G. (2014). Understanding the relationship between biotherapeutic protein stability and solid-liquid interfacial shear in constant region mutants of IgG1 and IgG4. J. Pharm. Sci..

[15] Tzannis S.T., Hrushesky W.J.M., Wood P.A., Przybycien T.M. (1997). Adsorption of a formulated protein on a drug delivery device surface. J. Colloid Interface Sci..

[16] Schmid M., Reichert K., Hammann F., Stäbler A. (2015). Storage time-dependent alteration of molecular interaction-property relationships of whey protein isolate-based films and coatings. J. Mater. Sci..

[17] Jesionowski T., Zdarta J., Krajewska B. (2014). Enzyme immobilization by adsorption: A review. Adsorption.

[18] Rafael C.R., Ángel B.M., Roberto F.L. (2011). Coupling chemical modification and immobilization to improve the catalytic performance of enzymes. Adv. Synth. Catal..

[19] Julia M.N., David T.P., Anneloes O.V., Francisco J. (2014). Immobilization of thermostable β-galactosidase on epoxy support and its use for lactose hydrolysis and galactooligosaccharides. World J. Microbiol. Biotechnol..

[20] Brady D., Jordaan J. (2009). Advances in enzyme immobilization. Biotechnol. Lett..

[21] Hwang E.T., Gu M.B. (2013). Enzyme stabilization by nano/microsized hybrid materials. Eng. Life Sci..

[22] Iyer P.V., Ananthanarayan L. (2008). Enzyme stability and stabilization-Aqueous and non-aqueous environment. Process Biochem..

[23] Betancor L., Luckarift H.R. (2008). Bioinspired enzyme encapsulation for biocatalysis. Trends Biotechnol..

[24] Mateo C., Palomo J.M., Fernandez-Lorente G., Guisan J.M., Fernandez-Lafuente R. (2007). Improvement of enzyme activity, stability and selectivity via immobilization techniques. Enzyme Microb. Technol..

[25] Barbosa O., Ortiz C., Berenguer-Murcia Á., Torres R., Rodrigues R.C., Fernandez-Lafuente R. (2015). Strategies for the one-step immobilization-purification of enzymes as industrial biocatalysts. Biotechnol. Adv..

[26] Zucca P., Sanjust E. (2014). Inorganic materials as supports for covalent enzyme immobilization: Methods and mechanisms. Molecules.

[27] Rodrigues R.C., Ortiz C., Berenguer-Murcia A., Torres R., Fernández-Lafuente R. (2013). Modifying enzyme activity and selectivity by immobilization. Chem. Soc. Rev..

[28] Santos A., Ladero M., García-Ochoa F. (1998). Kinetic Modeling of Lactose Hydrolysis by a β-Galactosidase from Kluyveromices Fragilis. Enzyme Microb. Technol..

[29] Nguyen T.T., Nguyen H.A., Arreola S.L., Mlynek G., Djinović-Carugo K., Mathiesen G., Nguyen T.H., Haltrich D. (2012). Homodimeric β-Galactosidase from Lactobacillus delbrueckii subsp. bulgaricus DSM 20081: Expression in Lactobacillus plantarum and Biochemical Characterization. J. Agric. Food Chem..

[30] Ladero M., Santos A., García J.L., García-Ochoa F. (2001). Activity over lactose and ONPG of a genetically engineered β-galactosidase from Escherichia coli in solution and immobilized: Kinetic modelling. Enzyme Microb. Technol..

[31] Fuchsbauer H.L., Gerber U., Engelmann J., Seeger T., Sinks C., Hecht T. (1996). Influence of gelatin matrices cross-linked with transglutaminase on the properties of an enclosed bioactive material using β-galactosidase as model system. Biomaterials.

[32] Kishore D., Kayastha A.M. (2012). Optimisation of immobilisation conditions for chick pea β-galactosidase (CpGAL) to alkylamine glass using response surface methodology and its applications in lactose hydrolysis. Food Chem..

[33] Lipowicz M., Basta N. (2014). 2014 Biopharma cold-chain forecast. Pharm. Commer..

[34] Bishara R.H. (2006). Cold chain management—An essential component of the global pharmaceutical supply chain. Am. Pharm. Rev..

[35] Abdallah A.A. (2013). Global pharmaceutical supply chain: A quality perspective. Int. J. Bus. Manag..

[36] Mariniello L., di Pierro P., Esposito C., Sorrentino A., Masi P., Porta R. (2003). Preparation and mechanical properties of edible pectin-soy flour films obtained in the absence or presence of transglutaminase. J. Biotechnol..

[37] Cao N., Fu Y., He J. (2007). Preparation and physical properties of soy protein isolate and gelatin composite films. Food Hydrocol..

[38] Zhang L., Chen P., Huang J., Yang G., Zheng L. (2003). Ways of strengthening biodegradable soy-dreg plastics. J. Appl. Polym. Sci..

[39] Anderson S.L., Grulke E.A., Delassus P.T., Smith P.B., Kocher C.W., Landes B.G. (1995). A Model for Antiplasticization in Polystyrene. Macromolecules.

[40] Chamarthy S.P., Pinal R. (2007). Moisture induced antiplasticization in microcrystalline cellulose compacts. Tablets Capsul..

[41] Vieira M.G.A., da Silva M.A., Santos L.O.D., Beppu M.M. (2011). Natural-based plasticizers and biopolymer films: A review. Eur. Polym. J..

[42] Gelin B.R., Karplus M. (1979). Side-chain torsional potentials: Effect of dipeptide, proteing and solvent environment. Biochemistry.

[43] Thomazine M., Carvalho R.A., Sobral P.J.A. (2005). Physical properties of gelatin films plasticized by blends of glycerol and sorbitol. J. Food Sci..

[44] Li N., Xue M.H., Yao H., Zhu J.J. (2005). Reagentless biosensor for phenolic compounds based on tyrosinase entrapped within gelatine film. Anal. Bioanal. Chem..

[45] Wadiak D.T., Carbonell R.G. (1975). Kinetic Behavior of Microencapsulated β-Galactosidase. Biotechnol. Bioeng..

